# An interrupted time series analysis of hospital admissions due to alcohol intoxication during the COVID‐19 pandemic in Tehran, Iran

**DOI:** 10.1038/s41598-022-14799-2

**Published:** 2022-06-22

**Authors:** Seyed Kaveh Hadeiy, Nasim Zamani, Rebecca McDonald, Omidvar Rezaei, Ali-Asghar Kolahi, Narges Gholami, Fariba Farnaghi, Hossein Hassanian-Moghaddam

**Affiliations:** 1 School of Medicine, Shahid Beheshti University of Medical Sciences, Tehran, Iran; 2 Social Determinants of Health Research Center, Shahid Beheshti University of Medical Sciences, Tehran, Iran; 3 Department of Clinical Toxicology, Loghman Hakim Hospital, School of Medicine, Shahid Beheshti University of Medical Sciences, South Karegar Street, Tehran, Iran; 4 SERAF, Norwegian Centre for Addiction Research, University of Oslo, Oslo, Norway; 5 Department of Neurosurgery, Loghman Hakim Hospital, Shahid Beheshti University of Medical Sciences, Tehran, Iran; 6 Department of Pediatrics, Loghman Hakim Hospital, School of Medicine, Shahid Beheshti University of Medical Sciences, Tehran, Iran

**Keywords:** Diseases, Health care, Risk factors

## Abstract

The COVID-19 outbreak affected mental health globally. One of the major concerns following the COVID-19 pandemic was increased incidence of risky behaviors including alcohol consumption. This study evaluates the trend of alcohol poisoning in Loghman-Hakim Hospital (LHH), the main referral center of poisoning in Tehran, during the 2-year period from 1 year prior to 1 year after the onset (February 23rd, 2020) of the COVID-19 epidemic in Iran. All patients admitted with alcohol intoxication from February 23rd, 2019 to February 22nd, 2021 were evaluated and patient data extracted from LHH electronic hospital records. Alcohols were categorized as toxic (methyl alcohol) and non-toxic (ethyl alcohol). Of 2483 patients admitted, 796/14,493 (5.49%) and 1687/13,883 (12.15%) had been hospitalized before and after the onset of the COVID-19 epidemic in Iran, respectively. In total, 140 patients did not survive, of whom 131 (93.6%) were confirmed to have methanol intoxication. Mortality was significantly higher during the outbreak (127 vs 13; P < 0.001; OR: 4.90; CI 95%: 2.75 to 8.73). Among the patients, 503 were younger than age 20. Trend of alcohol intoxication showed increases in children (57 vs 17) and adolescents (246 vs 183) when compared before and after the COVID-19 epidemic outbreak. A total of 955 patients were diagnosed with methanol toxicity which occurred more frequently during the COVID-19 era (877 vs 78; P < 0.001; OR: 10.00; CI 95%: 7.75 to12.82). Interrupted time series analysis (April 2016–February 2021) showed that in the first month of the COVID-19 epidemic (March 2020), there was a significant increase in the alcohol intoxication rate by 13.76% (P < 0.02, CI = [2.42–24.91]). The trend of alcohol intoxication as well as resulting mortality increased in all age groups during the COVID-19 epidemic in Iran, indicating urgent need for the prevention of high-risk alcohol use as well as improved treatment.

## Introduction

The COVID-19 pandemic influenced people’s mental health globally^1^. Anxiety, panic, post-traumatic stress disorder (PTSD), and depression were among the numerous detrimental effects of this pandemic on people’s minds^2^. The implemented strategies to control the infection have been based primarily on physical and social isolation like the closure of universities, schools and social events, mandatory facial mask use, ban of international travels and remote working^3,4^. These restrictive measures are shown to be related to the incidence of anxiety, depression, PTSD, sleep disorders, lack of self-control, and impaired self-esteem^5^. Media coverage of pandemic news has been another stressor to cause anxiety and fear in the population^6^. In addition, infected individuals have been exposed to an even higher incidence of mental disorders including depression, anxiety, and PTSD^7^. A cohort analysis revealed that almost one in five COVID 19 survivors was diagnosed with a psychiatric condition in 3 months after the infection^8^. A meta-analysis found that almost one-third of COVID 19 survivors experienced anxiety and depressive symptoms^9^. The mental consequences of the pandemic have been even more detrimental for the individuals with background mental disorders leading to the exacerbation of their conditions^10^. One of the consequences was increased risky behaviors including alcohol consumption and substance use^11^.

Alcohol use is believed to be the seventh leading risk factor for disability and premature death, and it is estimated that almost one-third of the global population drinks alcohol^12^. Alcohol consumption has been linked with vehicle accidents and related fatalities, domestic violence, suicide, and child abuse, all of which were therefore expected to occur more frequently during the COVID-19 pandemic^13,14^. Studies on the general population have found a connection between common mental disorders like anxiety and depression with binge drinking and alcohol use disorder^15–18^. In a study among college students, individuals with anxiety and depressive symptoms expressed a higher rate of weekly alcohol usage, and more adverse outcomes in comparison to individuals without symptoms^19^. In another study, anxiety and depression have been associated with 1.5 times more probability to alcohol abuse and 2.5 times to alcohol dependence^18^. A similar study found the same results in addition to the finding of PTSD to be related to two times higher probability of alcohol use disorder incidence^20^. Also, there is evidence of the association between increasing alcohol use and the worsening of mental health^21^. Coping with the stress is argued as a reason for alcohol consumption by motivational models as a mean for alleviation of the mental disorder symptoms^22,23^.

Reports show an increase in the trend of alcohol consumption throughout the world during the pandemic^24^. In Iran, there is no national center to monitor this trend. Additionally, alcohol consumption is prohibited in this country making the black market as the main source of provision of alcoholic beverages to the society and tracking a record on alcohol sale and use even more difficult. Along with criminalization due to the ban on alcohol in Iran, stigma is another factor that prevents individuals from seeking help and finding proper treatment; exposing them to more deleterious outcomes^25^.

This study evaluates the trend of alcohol intoxication (both toxic and non-toxic alcohols) in patients admitted to Loghman-Hakim Hospital (LHH), the main referral center for poisoned patients in the capital city of Tehran in the year preceding the COVID-19 outbreak and the year following its announcement in three different age groups of children, adolescents, and adults.

## Materials and methods

### Setting and study design

In a retrospective cross-sectional study, all alcohol-intoxicated patients admitted to LHH from February 23rd, 2019 to February 22nd, 2021 were enrolled into the study. February 23rd, 2020 was the date of announcement of COVID-19 epidemic in Iran (declared by the Iranian ministry of health) and was considered as the official date of COVID-19 initiation. LHH has the highest in-patient clinical toxicology admissions (25,000 patients/year) worldwide^26^.

### Age categorization

Patients aged 12 and younger were considered as children. The adolescent group consisted of the patients 13–20 years of age and all those 21 years and older were considered as adults.

### Type of alcohol

All patients with serum methanol level > 6.25 mmoL/L (20 mg/dL) were considered as methanol-intoxicated (either solely or in a co-ingestion with other medications/drugs/substances)^27^. Patients with an ethanol level higher than 50 mg/dL were considered as ethanol-poisoned again either solely or in combination with other medications/drugs/substances^28^.

### Epidemiological data and COVID-19 categorization

Patients’ demographic characteristics (age, gender), final diagnosis, and their final outcome were retrieved from the LHH electronic archive and analyzed.

### Missing data and statistical analysis

Data was analyzed using statistical package for social sciences (SPSS) software version 26 (IBM CO., Chicago, Ill, USA). Comparing parts with missing and non-missing data, it appears that missing data was completely random and randomly distributed across all observations. Chi-Square and Fisher’s exact tests were applied to search for significant differences in the categorical variables. The 95% confidence interval (CI) was used to estimate the precision of the odds ratio (OR). Mann–Whitney test applied to see differences among age and expressed as median [Inter quartile range]. Stata 14.0 (Stata Corp LLC, College Station, Texas, USA) was used for interrupted time series analysis to see whether the COVID-19 pandemic resulted in a shift in the level and trend of alcohol intoxication compared with those of the pre-COVID-19 period. We set the significance level at alpha = 0.05. For specific aim of time series analysis, data on the trend of alcohol intoxication was also gathered from April 2016 till February 2019 for a better assessment of pre-pandemic trend.

### Ethics approval and consent to participate

The study was approved by the Ethics Committee of Shahid Beheshti University of Medical Sciences, Tehran, Iran (IR.SBMU.RETECH.REC.1399.149). All experiments were in accordance with Helsinki declaration. Informed consent was obtained from all participants and/or if participants are under 16, from a parent and/or legal guardian.

## Results

### Epidemiological data

#### Age and gender

Of the 2483 patients referred to LHH with alcohol intoxication during the study period (out of a total of 28,376 patients hospitalized with poisoning), 1990 (80.1%) were male and 493 (19.9%) were female (Table 1). The mean (SD) age was 31.58 ± 13.76 (range: 1–87) years. A total of 796 out of 14,493 (32.1% of total alcohol cases of the study period and 5.49% of pre-COVID-19 cases) and 1687 out of 13,883 (67.9% total alcohol cases of the study period and 12.15% of during COVID-19 cases) had referred before and after the COVID-19 pandemic initiation in Iran. There were 361/14,493 (2.49%) vs. 474/13,883 (3.41%) deaths in hospitalized patients pre-COVID-19 vs. during COVID-19 respectively. Alcohol-related death was more common during COVID-19 period (127 vs. 13; P < 0.001; OR: 4.90; CI 95%: 2.75–8.73). Of the 796 patients referred before the infectious epidemic, 166 (20.8%) were female and 630 (79.2%) were male whereas in the 1687 admitted during the outbreak, 327 (19.4%) were female and 1360 (80.6%) were male. No significant difference was detected between the patients regarding their gender before and during the outbreak (P was nonsignificant).

**Table 1:** Number of alcohol intoxications and patients’ characteristics before and during COVID-19 outbreak (n = 2483).

Age group	Gender		Methanol n (%)	Ethanol n (%)	Co-ingestion n (%)	P1 (M^1^ vs. E^2^)	P2 (M vs. C^3^)	P3 (C vs.E)
Children (n = 74)	Female (n = 27)	Pre COVID (n = 6)	0	5 (83.7)	1(16.7)	0.278^‡^	0.125^‡^	0.300^‡^
		During COVID (n = 21)	7 (33.3)	14 (66.7)	0			
	Male (n = 47)	Pre COVID (n = 11)	1 (9.1)	10 (90.9)	0	0.078^‡^	–	–
		During COVID (n = 36)	14 (38.9)	22 (66.1)	0			
	Both (n = 74)	Pre COVID (n = 17)	1 (5.9)	15 (88.2)	1 (5.9)	0.028^‡^ 8.77* [1.08, 71.43]	0.087^‡^	0.308
		During COVID (n = 57)	21 (36.8)	36 (63.2)	0			
Adolescents 13–20 (n = 429)	Female (n = 87)	Pre COVID (n = 33)	2 (6.1)	15 (45.5)	16 (48.5)	0.007^‡^ 7.94* [1.58, 7.94]	0.003^‡^ 8.93* [6.29, 45.45]	0.805
		During COVID (n = 54)	19 (35.8)	17 (32.1)	17 (32.1)			
	Male (n = 342)	Pre COVID (n = 150)	10 (6.7)	78 (52)	62 (41.3)	< 0.001 4.38* [2.29, 10.53]	< 0.001 5.43* [0.071, 0.374]	0.468
		During COVID (n = 192)	52 (27.1)	84 (43.8)	56 (29.2)			
	Both (n = 429)	Pre COVID (n = 183)	12 (6.6)	93 (50.8)	78 (42.6)	< 0.001 5.35* [2.75, 10.52]	< 0.001 5.43* [2.67, 12.66]	0.465
		During COVID (n = 246)	71 (29.2)	101 (41.2)	73 (29.8)			
Adults > 20 (n = 1980)	Female (n = 379)	Pre COVID (n = 127)	10 (7.9)	41 (32.3)	76 (59.8)	< 0.001 8.20* [3.84, 17.54]	< 0.001 14.28* [6.94, 29.41]	0.034 1.74* [1.04, 2.91]
		During COVID (n = 252)	124 (49.2)	62 (24.6)	66 (26.2)			
	Male (n = 1601)	Pre COVID (n = 469)	50 (10.7)	265 (56.5)	154 (32.8)	< 0.001 11.23* [8.06, 15.62]	< 0.001 8.85* [6.21, 12.66]	12.5
		During COVID (n = 1132)	622 (54.9)	293 (25.9)	217 (19.2)			
	Both (n = 1980)	Pre COVID	60 (10.1)	306 (51.3)	230 (38.6)	< 0.001 10.75* [8.06, 15.62]	< 0.001 10.10* [7.35, 13.89]	0.637
		During COVID	746 (53.9)	355 (25.7)	283 (20.4)			
All ages (n = 2483)	Female (n = 493)	Pre COVID (n = 166)	12 (7.2)	61 (36.7)	93 (56)	< 0.001 8.13* [4.15, 15.87]	< 0.001 14.08* [7.25, 27.03]	0.015 1.73* [1.11, 2.88]
		During COVID (n = 327)	150 (46)	93 (28.5)	83 (25.5)			
	Male (n = 1990)	Pre COVID (n = 630)	61 (9.7)	353 (56)	216 (34.3)	< 0.001 10* [7.41, 13.51]	< 0.001 8.93* [6.49, 12.19]	0.339
		During COVID (n = 1360)	688 (50.6)	399 (29.3)	273 (20.1)			
	Both (n = 2483)	Pre COVID (n = 796)	73 (9.2)	414 (52)	309 (38.8)	< 0.001 9.61* [7.35, 12.66]	< 0.001 10* [7.52, 13.15]	0.747
		During COVID (n = 1687)	838 (49.7)	493 (29.2)	356 (29.1)			

^‡^Applying Fisher’s Exact test, *OR (95% CI), M^1^ (Methanol), E^2^ (Ethanol), C^3^ (Co-ingestion).

#### Alcohol type and mortality

**Figure 1:**
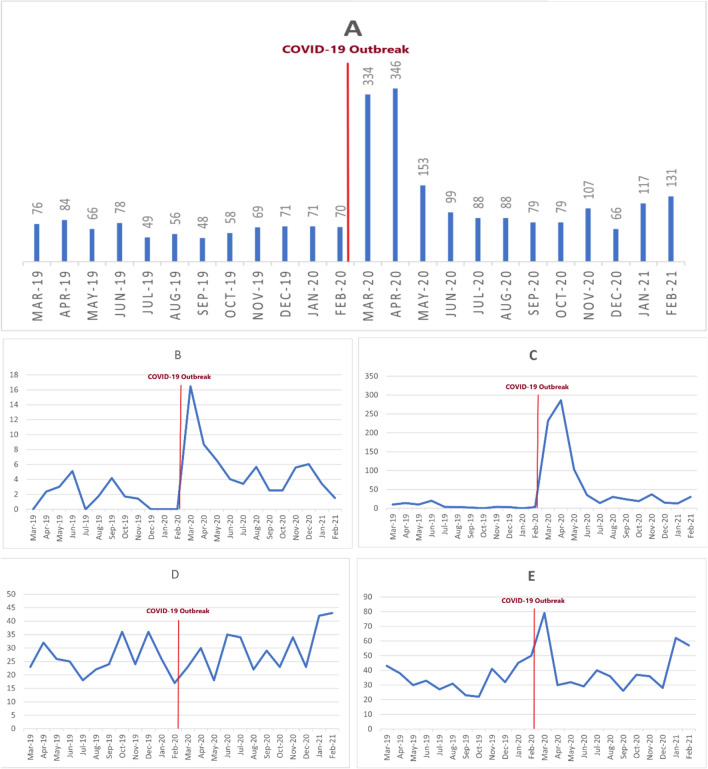
The trend of alcohol intoxication in the population: (**A**) all alcohols, (**B**) trend of mortality, (**C**) methanol, (**D**) co-ingestions, E ethanol.

In the 2-years scope of the study, 140 (5.64% of total alcohol cases and 16.77% of total mortalities due to poisoning) alcohol intoxicated patients died (21 females and 119 males). Mortality rates were 1.6% and 7.5% of alcohol intoxicated cases before and after the COVID-19 epidemic, respectively (13 versus 127 deaths; P < 0.001; OR = 4.90; CI 95% = 2.75–8.73). Figure 1 shows the trend of alcohol intoxication (Fig. 1A) and mortality (Fig. 1B) for each month in the period of the study. A total of 131 out of 140 (93.6%) non-survivors were intoxicated by methanol (solely or in combination with other medications/substances).

Of the total population of the study, 955 (38.5%) were diagnosed with methanol poisoning, either as a sole intoxicant (928; 37.4%) or with drugs/medications (27; 2.8%). Among 928 cases, 74 and 854 admitted before and after the COVID-19 epidemic respectively; P < 0.001; OR = 10.00; CI 95% = 7.73–12.94). Mortality was significantly higher among methanol-poisoned patients (P < 0.001; OR = 6.54; CI 95% = 4.35–9.84). Of 140 deaths, 131 (93.6%) had occurred among the patients diagnosed with methanol poisoning (mortality rate = 11.75%). Of this note, 5 patients (3.6%) consumed methanol in addition to at least one substance/medication.

**Figure 2:**
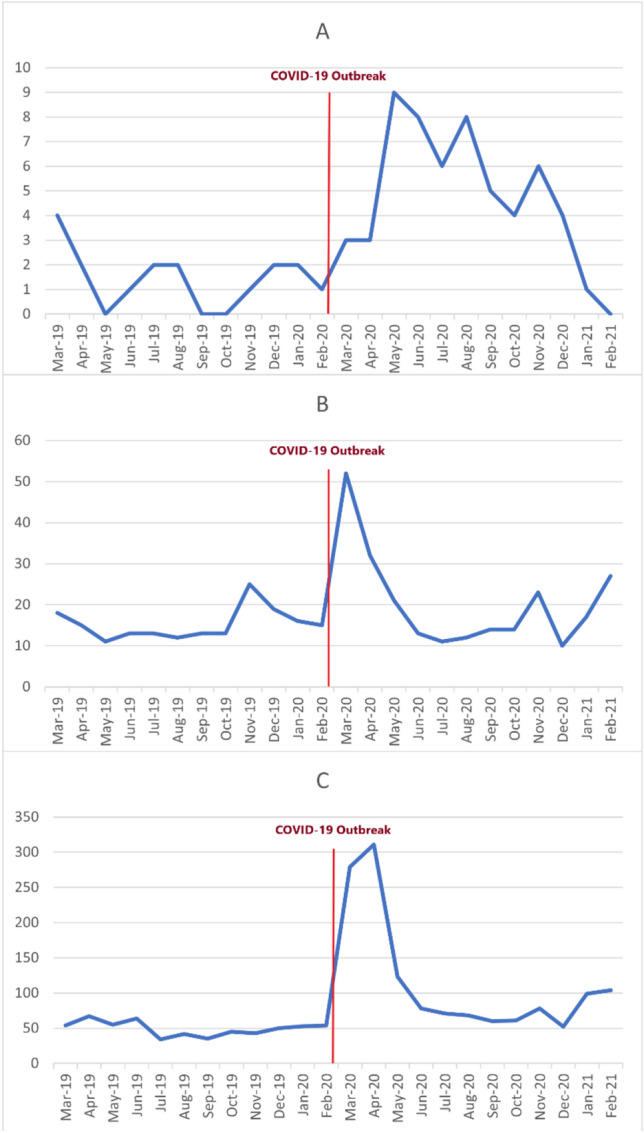
Trend of alcohol intoxication among children (**A**), adolescents (**B**), and Adults (**C**) Pre-COVID 19 and during COVID-19.

Almost 27% (665 patients) of our cases had been admitted with mixed poisoning of alcohols and other substances/medications, of whom 44 and 621 were finally diagnosed with methanol and ethanol intoxication as the alcohol involved in the intoxication, again with no significant difference between the pre- and during-COVID epidemic. Table 2 shows the pair comparisons of three groups together. Mortality was significantly higher before and during-COVID epidemic while methanol was compared to ethanol (P = 0.016; OR = 29.41; CI 95% = 2.41–333.3) or to the patients who had co-ingestion (P = 0.045; OR = 9.71; CI 95% = 7.25–13.18). No significant differences were found between ethanol and co-ingestion group in alive or dead cases any time. Only 11 patients with mixed poisoning did not survive (mortality rate = 1.65%). Figure 1C,D depict the trend of poisoning with methanol and mixed poisoning during the study period, respectively. Trend of alcohol intoxication among children (A), adolescents (B), and Adults (C) Pre-COVID 19 and during COVID-19 are shown in Fig. 2.

**Table 2:** Comparison of different groups of alcoholic ingestions 1 year before and after COVID-19 (n = 2483).

	Survived (n = 2703)		Death (n = 140)	
	Pre COVID-19 (n = 783)	During COVID-19 (n = 1560)	Pre COVID-19 (n = 13)	During COVID-19 (n = 127)
Methanol n (%)	65 (2.40)	720 (26.64)	8 (5.71)	118 (84.28)
Ethanol n (%)	414 (15.3)	492 (18.20)	2 (1.43)	1 (0.71)
P-value	< 0.001		0.016	
OR (95% CI)	9.26 (12.34, 6.99)		29.41 (2.41, 333.33)	
Co-ingestion n (%)	306 (11.32)	348 (12.87)	3 (2.14)	8 (5.71)
Ethanol n (%)	414 (15.31)	492 (18.20)	1 (0.71)	2 (1.43)
P-value OR (95% CI)	0.643		0.505	
Methanol n (%)	65 (2.40)	720 (26.64)	8 (5.71)	118 (84.28)
Co-ingestion n (%)	306 (11.32)	348 (12.87)	3 (2.14)	8 (5.71)
P-value	< 0.001		0.045	
OR (95% CI)	5.52 (1.22, 25.00)		9.71 (7.25, 13.18)

### Interrupted time series analysis (April 2016–February 2021)

#### Alcohol intoxication

**Figure 3:**
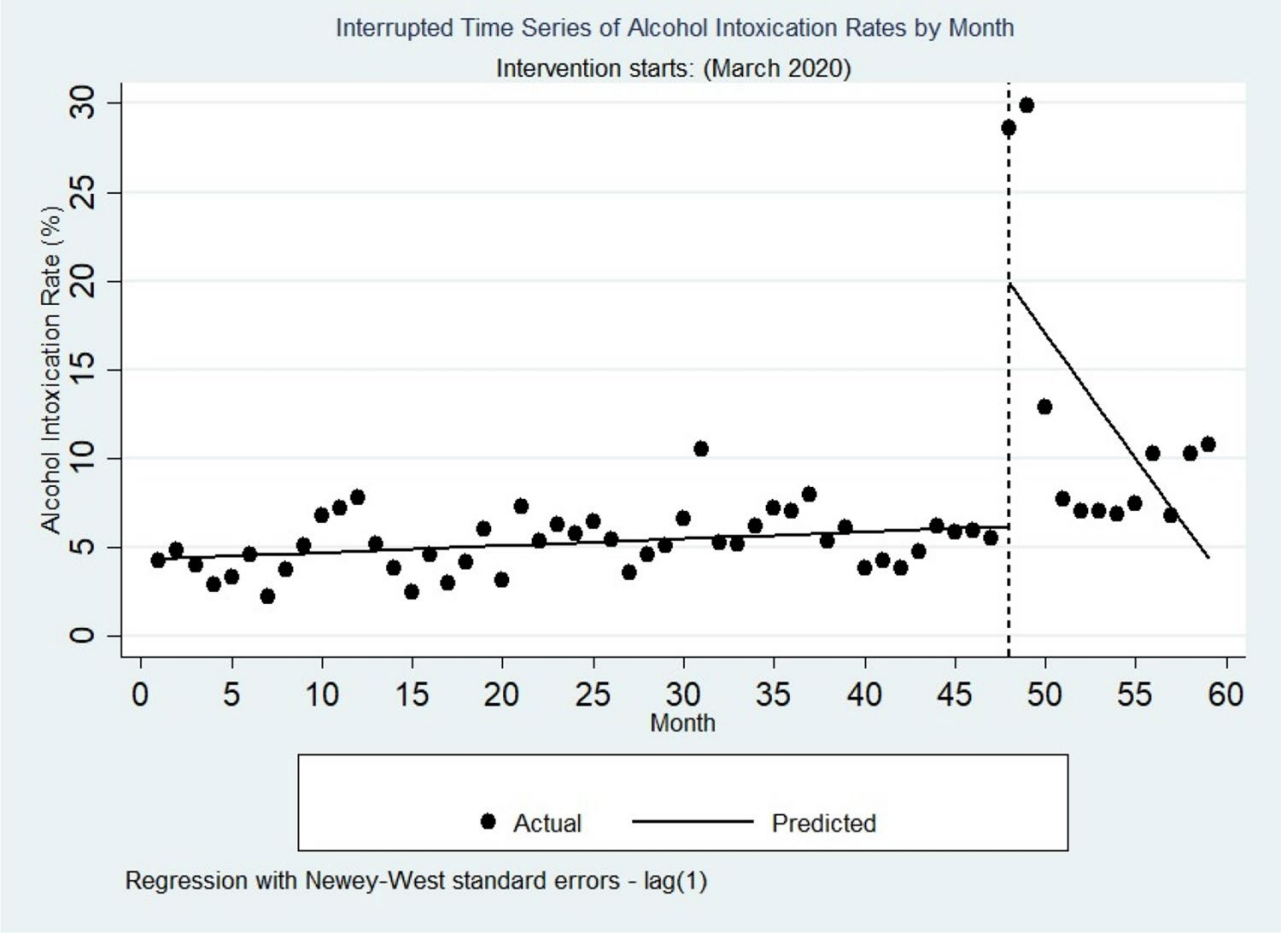
Interrupted time series of alcohol intoxication rates by month.

As shown in Table 3 and Fig. 3, the alcohol intoxication rate increased every month before March 2020 by 0.04%. This increase was statistically significant (P = 0.03, CI = [0.01–0.07]).

**Table 3:** Interrupted time series analysis comparing level, trend and mortality of alcohol intoxication in pre-COVID-19 to during COVID-19 period.

Variable	Coef	SE	P	95% CI
**Alcohol intoxication**				
cons	4.34	0.49	< 0.001	3.35 to 5.34
_t	0.04	0.02	0.03	0.01 to 0.07
_x48	13.67	5.61	0.02	2.42 to 24.91
_x_t48	− 1.44	0.73	0.05	− 2.91 to 0.02
Treated	− 1.41	0.73	0.06	− 2.84 to 0.02
**Alcohol-related mortality**				
cons	1.78	0.50	0.001	0.77 to 2.79
_t	− 0.01	0.02	0.47	− 0.05 to 0.02
_x48	8.50	2.47	0.001	3.54 to 13.47
_x_t48	− 0.73	0.31	0.02	− 1.35 to − 0.10
Treated	− 0.74	0.31	0.02	− 1.36 to − 0.12

In the first month of the COVID-19 pandemic (March 2020), there appeared to be a significant increase in the alcohol intoxication rate of 13.76% (P < 0.02, CI = [2.42–24.91]).

The trend of alcohol intoxication after the COVID-19 pandemic (relative to the pre-COVID-19 trend) decreased by 1.44% per month, which was not statistically significant (P = 0.05, CI = [− 2.91 to 0.02]).

In addition, after the COVID-19 pandemic, alcohol intoxication decreased by 1.41% per month. This decrease was not statistically significant (P = 0.06, CI = [− 2.84 to 0.02]).

#### Alcohol-related mortality

**Figure 4:**
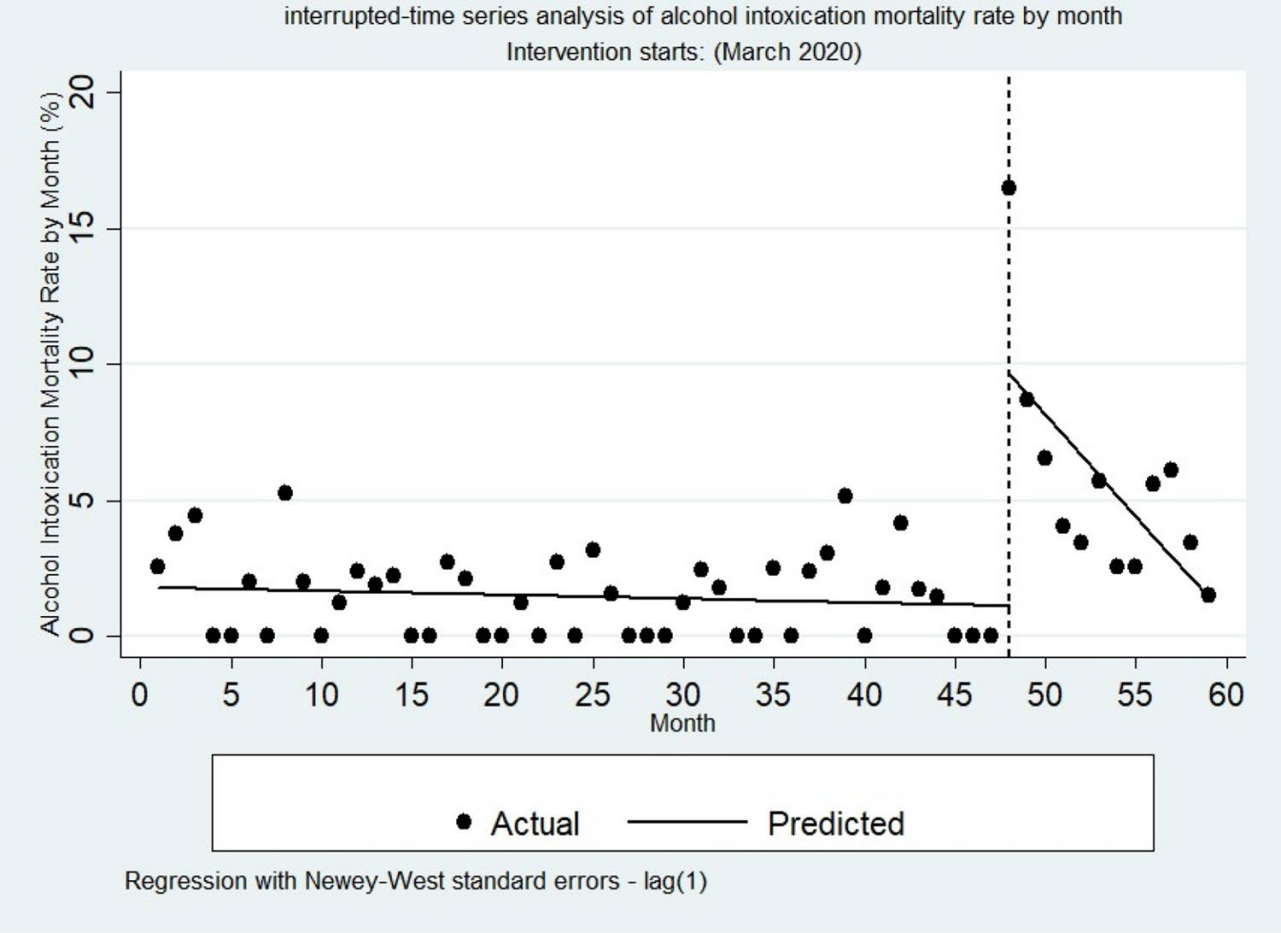
Interrupted time series of alcohol-related mortalities by month.

As shown in Table 3 and Fig. 4, the alcohol-related mortality rate decreased every month before March 2020 by 0.01%. This decrease was not statistically significant (P = 0.47, CI = [− 0.05 to 0.02]).

In the first month of the COVID-19 pandemic (March 2020), there appeared to be a significant increase in the alcohol intoxication mortality rate of 8.50% (P = 0.001, CI = [3.54 to 13.47]). The trend of alcohol intoxication after the COVID-19 pandemic (relative to the pre-COVID-19 trend) decreased by 0.73% per month, which was statistically significant (P = 0.02, CI = [− 1.35 to − 0.10]). In addition, after the COVID-19 pandemic, alcohol intoxication decreased by 0.74% per month. This decrease was statistically significant (P = 0.02, CI = [− 1.36 to − 0.12]).

## Discussion

As far as we know, this study reports the highest number of methanol poisoning cases in a single center in the world addressing the trend of alcohol intoxication before and after COVID-19 pandemic^29,30^. Based on our results, a rough twofold increase is detected in the number of alcohol poisoned cases during the pandemic. In addition, there was a discernible rise in the first 3 months after the beginning of the outbreak. During the pandemic, people experienced numerous adversities in their lives including lockdowns and social distancing, distant working, unemployment, loss of the family, and school closures^31^. During the early days of the pandemic, it was challenging for many people to come up with a variety of stressful stimuli including the fear of the disease and uncertainty about the future^32^. Studies have reported increased anxiety and distress in several countries^33,34^. Increasing alcohol consumption was a coping behavior to this elevated anxiety^32^. Evidence shows dramatic increase in the alcoholic beverages sales^35,36^ even though several countries utilized restrictive measures to control alcohol consumption^24^.

Working from home needs adjustment in daily routines and consequently causes an elevated anxiety state^34^. Unemployment acted similarly and resulted in higher alcohol consumptions^32^. Evidence from former economic crises also highlights similar consequences^37^. In such situation, despite being unemployed, people may tend to use cheap alcoholic beverages in a cost-saving manner^38^. Solitary drinking is also believed to be a risk factor for deleterious outcomes of alcohol consumption^39^.

Additionally, patients with COVID-19 infection and their families as well as the health care workers experience stigmatizations resulting in stressful stimuli^40^. There are reports of concomitant COVID-19 infection and methanol poisoning^41^. Loss of friends and relatives and complicated grief are of the other factors altering alcohol consumption behavior^42^. Having to provide childcare while working from home also reported to be stressful for the parents during the pandemic^29,31^. Lockdown and restrictive measures have also made school and university student another vulnerable group; it shown that interactions with teachers and classmates have a fundamental role in reducing mental health problems including anxiety and depression^43^.

The pandemic has also been associated with an increase in depression in the public^44^. Many people with psychological and mental disorders may be deprived from obtaining appropriate care due to the fear of becoming infected or reduced resources due to restrictive measures^31^.

In crises, people seek for new information to be up to date from any source possible which may predispose the society to the danger of false information and rumors^2^. Based on our results, during the first 3 months of the outbreak, a discernible rise was spotted in the number of alcohol-poisoned cases and specifically in the numbers of methanol-poisoned patients. It was erroneously propagated that alcohol prevented COVID-19 infection. People started to drink alcoholic beverages and even sanitizers to prevent infection. On the other hand, increased demand for hand sanitizers led to scarcity and finally indulgence of the market with low-quality products containing methanol^45^.

The increased risk of poisoning was detected in all age groups. It was initially believed that alcohol poisoning was more suicidal or deliberate among adults and adolescents and more accidental among children^46^. However, our results showed that even in the adults it was more recreational. This could also be due to stocking of low-quality alcohol-containing products and sanitizers at homes.

In Iran, alcohol drinking is prohibited, and the black market provides alcoholic beverages to the public. There is no national center for monitoring alcohol consumption in the society, and data on its sale and use are missing. However, a recent study suggests the rising trend of alcohol poisoning through the recent years^47^. Outbreaks of methanol poisoning in Iran have been occurred frequently and have been always a public health concern^48–50^. Our data suggests a sharp rise in methanol poisoning in the first 3 months after the outbreak resulting in high mortality in the total population and specifically among adolescents. Of seven deceased adolescents, methanol poisoning was diagnosed in six, all happening in those 3 months. Another fact is that methanol poisoning regularly peaked during the year preceding the COVID-19 epidemic but after that it never reached a zero level. This is another concern implying the fact that the market is continuously indulged with low-quality products resulting in ongoing methanol poisoning.

Due to the prohibition of alcohol consumption and legal consequences, intoxicated people usually avoid referring to the health care facilities until the final stages of the poisoning resulting in a higher mortality and morbidity^51^. This emphasizes the importance of active case finding^52^.

### Limitations

Considering the retrospective nature of the study, we could not retrieve the intent of alcohol ingestion in many cases; also, we were not able to follow the patients after discharge to check for possible mortality post-discharge. The generalizability of this single-center study is unclear. The current study may be subject to selection bias, as we did not measure methanol and ethanol levels of all poisoned patients. Measurement was done only in those with a history of alcohol ingestion or clinical finding/lab exams in favor of alcohol intoxication.

## Conclusion

Market surveillance and increasing public awareness should be intensified to prevent further methanol poisonings during the COVID-19 epidemic in Iran. Public education on dangerous effects of non-standard alcoholic beverages and sanitizers as well as proper stocking of these products at home can decrease alcohol poisonings in all age groups.

Measures to control alcohol consumption and suicidal attempts should be followed more intensely among adolescents and social support should be provided for this age group especially during crises of this kind as more risky behaviors are anticipated in them^53^. This is extremely important as their drinking habit in this period may affect their drinking habit as an adult^54,55^.

It should be borne in mind that monitoring and measures should not be limited to the pandemic era. Previous experiences from the SARS epidemic^55^ and the economic crisis of 2007–2008^56^ showed a delayed increase in the trend of alcohol consumption in the following years which should be foreseen and avoided by implementation of adequate preventive measures.

## Data Availability

The datasets generated and/or analyzed during the current study are available from the corresponding author on reasonable request.

## Abbreviations

CI: Confidence Interval
GCS: Glasgow Coma Scale
HD: Hemodialysis
OR: Odds Ratio
SPSS: Statistical Package for the Social Sciences
VDs: Visual disturbances
WHO: World Health Organization

## References

[1] Torales J, O'Higgins M, Castaldelli-Maia JM, Ventriglio A (2020). The outbreak of COVID-19 coronavirus and its impact on global mental health. Int. J. Soc. Psychiatry.

[2] Shuja KH, Aqeel M, Jaffar A, Ahmed A (2020). COVID-19 pandemic and impending global mental health implications. Psychiatr. Danub..

[3] Han E, Tan MMJ, Turk E (2020). Lessons learnt from easing COVID-19 restrictions: An analysis of countries and regions in Asia Pacific and Europe. Lancet.

[4] Hale, T., Webster, S., Petherick, A. *et al*. Oxford COVID-19 government response tracker [Internet]. Blavatnik School of Government. Data use policy: Creative commons attribution CC BY standard (2021). https://covidtracker.bsg.ox.ac.uk/. Accessed 29 Jan, 2021.

[5] Hossain, M. M., Sultana, A., Purohit, N. Mental health outcomes of quarantine and isolation for infection prevention: A systematic umbrella review of the global evidence. PsyArXiv. 10.31234/osf.io/dz5v2 (2020). 10.4178/epih.e2020038 PMC7644933 32512661

[6] Rubin GJ, Wessely S (2020). The psychological effects of quarantining a city. BMJ.

[7] Mendez R, Balanza-Martınez V, Luperdi SC (2021). Short-term neuropsychiatric outcomes and quality of life in COVID-19 survivors. J. Intern. Med..

[8] Taquet M, Luciano S, Geddes JR, Harrison PJ (2020). Bidirectional associations between COVID-19 and psychiatric disorder: Retrospective cohort studies of 62 354 COVID-19 cases in the USA. Lancet Psychiatry.

[9] Kong, X., Zheng, K., Tang, M. *et al*. Prevalence and factors associated with depression and anxiety of hospitalized patients with COVID-19. *medRxiv*. 10.1101/2020.03.24.20043075 (2020).

[10] Ostuzzi G, Papola D, Gastaldon C (2020). Safety of psychotropic medications in people with COVID-19: Evidence review and practical recommendations. BMC Med..

[11] Gunnell D, Appleby L, Arensman E (2020). Suicide risk and prevention during the COVID-19 pandemic. Lancet Psychiatry.

[12] Griswold MG, Fullman N, Hawley C, Arian N, Zimsen SRM, Tymeson HD (2018). Alcohol use and burden for 195 countries and territories, 1990–2016: A systematic analysis for the global burden of disease study 2016. Lancet.

[13] White AM, Castle IP, Hingson RW, Powell PA (2020). Using death certificates to explore changes in alcohol-related mortality in the United States, 1999 to 2017. Alcohol Clin. Exp. Res..

[14] World Health Organization (2020). Alcohol and COVID-19: What You Need to Know.

[15] Paljärvi T, Koskenvuo M, Poikolainen K, Kauhanen J, Sillanmäki L, Mäkelä P (2009). Binge drinking and depressive symptoms: A 5-year population-based cohort study. Addiction.

[16] Lee YY, Wang P, Abdin E, Chang S, Shafie S, Sambasivam R (2020). Prevalence of binge drinking and its association with mental health conditions and quality of life in Singapore. Addict. Behav..

[17] Nazareth I, Walker C, Ridolfi A, Aluoja A, Bellon J, Geerlings M (2011). Heavy episodic drinking in Europe: A cross section study in primarycare in six European countries. Alcohol Alcohol..

[18] Lai HMX, Cleary M, Sitharthan T, Hunt GE (2015). Prevalence of comorbid substance use, anxiety and mood disorders in epidemiological surveys, 1990–2014: A systematic review and meta-analysis. Drug Alcohol Depend..

[19] Austin MA, Villarosa-Hurlocker MC (2021). Drinking patterns of college students with comorbid depression and anxiety symptoms: The moderating role of gender. J. Subst. Abuse.

[20] Puddephatt JA, Irizar P, Jones A, Gage SH, Goodwin L (2022). Associations of common mental disorder with alcohol use in the adult general population: A systematic review and meta-analysis. Addiction.

[21] Jane-Llopis E, Matytsina I (2006). Mental health and alcohol, drugs and tobacco: A review of the comorbidity between mental disorders and the use of alcohol, tobacco and illicit drugs. Drug Alcohol Rev..

[22] Wills TA, Shiffman S (1985). Coping and substance use: A conceptual framework. Coping Subst. Use.

[23] Khantzian E (1997). The self-medication hypothesis of substance use disorders: A reconsideration and recent applications. Harv. Rev. Psychiatry.

[24] Sugarman DE, Greenfield SF (2021). Alcohol and COVID-19: How do we respond to this growing public health crisis?. J. Gen. Intern. Med..

[25] Aqeel M, Gul M (2020). Acceptance and commitment therapy for treatment of stigma and shame in substance use disorders: A double-blind, parallel-group, randomized controlled trial. J. Subst. Use..

[26] Hassanian-Moghaddam H (2013). An educational and research opportunity for the largest university hospital poison control centers; Tehran and Cairo. Egypt. J. Forensic Sci..

[27] Hassanian-Moghaddam H, Zamani N (2016). A brief review on toxic alcohols: Management strategies. Iran. J. Kidney Dis..

[28] Peng SH, Hsu SY, Kuo PJ, Rau CS, Cheng YA, Hsieh CH (2016). Influence of alcohol use on mortality and expenditure during hospital admission: A cross-sectional study. BMJ Open.

[29] Hassanian-Moghaddam H, Zamani N, Kolahi AA, McDonald R, Hovda KE (2020). Double trouble: Methanol outbreak in the wake of the COVID-19 pandemic in Iran—A cross-sectional assessment. Crit. Care.

[30] Mousavi-Roknabadi RS, Arzhangzadeh M, Safaei-Firouzabadi H, Mousavi-Roknabadi RS, Sharifi M, Fathi N, Zarei Jelyani N, Mokdad M (2022). Methanol poisoning during COVID-19 pandemic; A systematic scoping review. Am. J. Emerg. Med..

[31] Zalsman G, Stanley B, Szanto K, Clarke DE, Carli V, Mehlum L (2020). Suicide in the time of COVID-19: Review and recommendations. Arch. Suicide Res..

[32] Wardell JD, Kempe T, Rapinda KK (2020). Drinking to cope during COVID-19 pandemic: The role of external and internal factors in coping motive pathways to alcohol use, solitary drinking, and alcohol problems. Alcohol Clin. Exp. Res..

[33] Centers for Disease Control and Prevention. Mental health household pulse survey [Web site]. June 10, 2020 (2020). https://www.cdc.gov/nchs/covid19/pulse/mental-health.htm Accessed June 10, 2020.

[34] Centre for Addiction and Mental Health. COVID-19 national survey dashboard [Web site]. (2020). https://www.camh.ca/en/healthinfo/mental-health-and-covid-19/covid-19-national-survey. Accessed June 8, 2020.

[35] Bakaloudi DR, Jeyakumar DT, Jayawardena R, Chourdakis M (2021). The impact of COVID-19 lockdown on snacking habits, fast-food and alcohol consumption: A systematic review of the evidence. Clin. Nutr..

[36] Lee BP, Dodge JL, Leventhal A, Terrault NA (2021). Retail alcohol and tobacco sales during COVID-19. Ann. Intern. Med..

[37] de Goeij MC, Suhrcke M, Toffolutti V, van de Mheen D, Schoenmakers TM, Kunst AE (2015). How economic crises affect alcohol consumption and alcohol-related health problems: A realist systematic review. Soc. Sci. Med..

[38] Rehm J, Kilian C, Ferreira-Borges C (2020). Alcohol use in times of the COVID 19: Implications for monitoring and policy. Drug Alcohol Rev..

[39] Skrzynski CJ, Creswell KG (2020). Associations between solitary drinking and increased alcohol consumption, alcohol problems, and drinking to cope motives in adolescents and young adults: A systematic review and meta-analysis. Addiction.

[40] Shigemura J, Ursano RJ, Morganstein JC, Kurosawa M, Benedek DM (2020). Public responses to the novel 2019 coronavirus (2019-nCoV) in Japan: Mental health consequences and target populations. Psychiatry Clin. Neurosci..

[41] Zamani N, Gheshlaghi F, Haghighi-Morad M, Bahrami-Motlagh H, Alavi Darazam I, Hadeiy SK, McDonald R, Hassanian-Moghaddam H (2021). Prevalence of clinical and radiologic features in methanol-poisoned patients with and without COVID-19 infection. Acute Med. Surg..

[42] Aoyama M, Sakaguchi Y, Fujisawa D (2020). Insomnia and changes in alcohol consumption: Relation between possible complicated grief and depression among bereaved family caregivers. J. Affect. Disord..

[43] Aqeel M, Rehna T, Shuja KH, Abbas J (2022). Comparison of students' mental wellbeing, anxiety, depression, and quality of life during COVID-19's full and partial (smart) lockdowns: A follow-up study at a 5-month interval. Front. Psychiatry.

[44] Wang C, Pan R, Wan X (2020). Immediate psychological responses and associated factors during the initial stage of the 2019 coronavirus disease (COVID-19) epidemic among the general population in China. Int. J. Environ. Res. Public Health.

[45] Mahdavi SA, Kolahi AA, Akhgari M (2021). COVID-19 pandemic and methanol poisoning outbreak in Iranian children and adolescents: A data linkage study. Alcohol Clin. Exp. Res..

[46] Hadeiy SK, Parhizgar P, Hassanian-Moghaddam H (2021). Trends of acute drug and chemical toxicities in adults and adolescents in Tehran, Iran between 2012 and 2018: A retrospective chart review. Drug Chem. Toxicol..

[47] Delirrad M, Mohammadi AB (2020). New methanol poisoning outbreaks in Iran following COVID-19 pandemic. Alcohol Alcohol..

[48] Aghababaeian H, Araghi Ahvazi L, Ostadtaghizadeh A (2019). The methanol poisoning outbreaks in Iran 2018. Alcohol Alcohol..

[49] Massoumi G, Saberi K, Eizadi-Mood N, Shamsi M, Alavi M, Morteza A (2012). Methanol poisoning in Iran, from 2000 to 2009. Drug Chem. Toxicol..

[50] Yazdi-Feyzabadi V, Mehrolhassani MH, Zolala F, Haghdoost A, Oroomiei N (2019). Determinants of risky sexual practice, drug abuse and alcohol consumption in adolescents in Iran: A systematic literature review. Reprod. Health.

[51] Hassanian-Moghaddam H, Nikfarjam A, Mirafzal A (2015). Methanol mass poisoning in Iran: Role of case finding in outbreak management. J. Public Health (Oxf)..

[52] Blumenthal H, Ham LS, Cloutier RM, Bacon AK, Douglas ME (2016). Social anxiety, disengagement coping, and alcohol-use behaviors among adolescents. Anxiety Stress Coping.

[53] Wittchen HU, Behrendt S, Höfler M (2008). What are the high risk periods for incident substance use and transitions to abuse and dependence? Implications for early intervention and prevention. Int. J. Methods Psychiatr. Res..

[54] Deas D, Riggs P, Langenbucher J, Goldman M, Brown S (2000). Adolescents are not adults: Developmental considerations in alcohol users. Alcohol Clin. Exp. Res..

[55] Wu P, Liu X, Fang Y (2008). Alcohol abuse/dependence symptoms among hospital employees exposed to a SARS outbreak. Alcohol Alcohol..

[56] Manthey J, Shield KD, Rylett M, Hasan OSM, Probst C, Rehm J (2019). Global alcohol exposure between 1990 and 2017 and forecasts until 2030: A modelling study. Lancet.

